# *In Vitro* Differential Diagnosis of Clavus and Verruca by a Predictive Model Generated from Electrical Impedance

**DOI:** 10.1371/journal.pone.0093647

**Published:** 2014-04-04

**Authors:** Chien-Ya Hung, Pei-Lun Sun, Shu-Jen Chiang, Fu-Shan Jaw

**Affiliations:** 1 Institute of Biomedical Engineering, National Taiwan University, Taipei, Taiwan; 2 Department of Dermatology, Mackay Memorial Hospital, Taipei, Taiwan; 3 Institute of Zoology, National Taiwan University, Taipei, Taiwan; University Hospital Hamburg-Eppendorf, Germany

## Abstract

**Background:**

Similar clinical appearances prevent accurate diagnosis of two common skin diseases, clavus and verruca. In this study, electrical impedance is employed as a novel tool to generate a predictive model for differentiating these two diseases.

**Materials and Methods:**

We used 29 clavus and 28 verruca lesions. To obtain impedance parameters, a LCR-meter system was applied to measure capacitance (*C*), resistance (*R_e_*), impedance magnitude (*Z*), and phase angle (*θ*). These values were combined with lesion thickness (*d*) to characterize the tissue specimens. The results from clavus and verruca were then fitted to a univariate logistic regression model with the generalized estimating equations (GEE) method. In model generation, log *Z_SD_* and *θ_SD_* were formulated as predictors by fitting a multiple logistic regression model with the same GEE method. The potential nonlinear effects of covariates were detected by fitting generalized additive models (GAM). Moreover, the model was validated by the goodness-of-fit (GOF) assessments.

**Results:**

Significant mean differences of the index *d, R_e_, Z,* and *θ* are found between clavus and verruca (*p*<0.001). A final predictive model is established with *Z* and *θ* indices. The model fits the observed data quite well. In GOF evaluation, the area under the receiver operating characteristics (ROC) curve is 0.875 (>0.7), the adjusted generalized *R* ^2^ is 0.512 (>0.3), and the *p* value of the Hosmer-Lemeshow GOF test is 0.350 (>0.05).

**Conclusions:**

This technique promises to provide an approved model for differential diagnosis of clavus and verruca. It could provide a rapid, relatively low-cost, safe and non-invasive screening tool in clinic use.

## Introduction

Verruca and clavus are two skin disorders commonly encountered in dermatological clinics. Verruca, also referred to as wart, is an infection by human papillomaviruses. Based on the involved site and morphology, verruca can be categorized into several clinical forms, such as verruca vulgaris, plantar and palmar warts, verruca plana, anogenital warts, condyloma, etc. [1]. The former two forms are far more common than the others. These two forms are single or multiple keratotic spiny papules or nodules on hands and/or feet. They are symptomless but can cause pain when grow endophytically on soles. Punctate black dots in verruca, which result from thrombosed capillaries, can be observed by dermoscopy. Verruca lesions may increase in size and number with time and can be contagious.

Although verruca and clavus resemble each other in clinical symptoms, appearances and predilection sites, the latter unlike the former is not contagious. Clavus results from prolonged pressure and friction on the skin [2]. Clavus lesions are painful hard keratotic papules and nodules on soles and palms. Under a dermoscope, a clavus has a compact, homogeneous translucent central core. There may be some hemorrhages resulting from ruptured capillaries due to shearing forces on skin. No punctate black dots are observed in clavus (Figure S1).

Differential diagnosis between verruca and clavus is mainly based on etiology, pathogenesis, treatment, and means of prevention. The standard treatment for verruca is liquid nitrogen cryotherapy. Other therapeutic modalities include curettage, surgical excision, chemical caustics, and immunotherapies. On the other hand, paring of central radix of corn and topical keratolytics are used in treating clavus [1], [2]. Histopathology is of great diagnostic value for these two diseases. In typical verruca, there are hyperkeratosis, parakeratosis and acanthosis of epidermis with koilocytes at the upper epidermis and dilated, thrombosed dermal capillaries. As to clavus, there is a prominent parakeratotic plug in the stratum corneum with an underlying atrophied stratum Malpighian layer. Although dermoscopy is a useful non-invasive tool to make the differential diagnosis, its clinical application is operator-dependent, especially in those cases where typical features are lacking. Therefore, finding an effective alternative approach for differentiating between clavus and verruca is highly desirable for clinical diagnosis.

Electrical impedance, known as a rapid, safe tool with a relatively low cost, has recently been used in measuring skin or stratum corneum hydration [3]–[12]. It has also been applied to distinguish allergic and irritant contact dermatitis by evaluating the degree of irritation in human skin [13]–[15]. In addition, the electrical impedance values have been assessed to discriminate skin tumors, such as melanoma, dysplastic nevi, nodular basal cell carcinoma, superficial basal cell carcinoma, as well as benign and malignant skin lesions [16]–[22]. Clavus has a much thicker stratum corneum and a thinner epidermis than verruca. It is thus expected that their differential thickness and capacitance of stratum corneum will cause differences in their electrical impedance, which can be used as a predictive tool for diagnosing the two types of skin disorders.

In this study, we employed electrical impedance to differentiate between clavus and verruca. The electrical properties of target tissues were measured after a small current or voltage had been applied to them. The resulting impedance values reflected well the different histological constituents of clavus and verruca. It appears quite promising that our study will help build an electrical impedance system for clinical use in the future to quickly and unequivocally discern between clavus and verruca.

## Materials and Methods

On the basis of dermoscopic features, specimens were diagnosed and collected by dermatologists from clavus and verruca patients at out-patient clinic. A total of 57 lesions obtained from hands or feet of 29 patients consisted of 29 clavus and 28 verruca lesions. The criteria for a valid specimen were as follows: Each specimen was (1) diagnosed unequivocally under a dermoscope, (2) greater than 3 mm in diameter, (3) collected from a patient whose lesion has not been treated before, and, (4) regarded as independent if more than one lesion was removed from the same patient. All the samples were processed in accordance with standard treatment protocols for clavus and verruca at our hospital, *i.e.* paring the outer part of the lesions, and applying triacetic acid solution and liquid nitrogen cryotherapy on clavus and verruca, respectively. All the samples were subjected to impedance measuring without any patient information, except the diagnosis. After measuring, all the samples were discarded and disinfected. No patient medical profiles were recorded, thus no linkage between the patients and the samples could be retrieved thereafter.

### Impedance measurement

To assemble an electrical impedance system, both sides of each specimen were sealed by two pieces of insulation tape, each with a 3 mm hole in the center. Two holes on each side of the insulation tape were allocated on opposing sides. Therefore, a controlled area of the specimen was used for measurement. This was followed by attaching a pair of pre-gelled electrodes (MEDI-TRACE Mini, Kendall/Tyco, USA) to the two tape holes. Next, the sample was placed in a shielding chamber to reduce noises. Its electrical properties were then measured by a commercial LCR-meter (LCR-821, Instek, Taiwan) under 1 V, at the frequencies of 50 Hz, 80 Hz, 100 Hz, 200 Hz, 1 kHz, 2 kHz, 5 kHz, 10 kHz, 100 kHz, and 1 MHz. The data acquisitions were immediately performed after setup, at 30 min and 60 min. The impedance indices include capacitance (*C*), resistance (*R_e_*), impedance magnitude (*Z*), and phase angle (*θ*). Additionally, the thickness (*d*) of the sample was measured by vernier caliper. Temperature and relative humility were recorded.

### Statistics analysis

Statistical analysis of the electrical impedance system was performed using the R 3.0.2 software (R Foundation for Statistical Computing, Vienna, Austria) [23]. In statistical testing, two-sided *p* value ≤0.05 was considered statistically significant. To compare the differentiating powers across frequencies, all observations from 10 frequencies were standardized and examined by conditional plots. After scrutinizing the differences in the conditional plots at 10 frequencies, and also referring to previous studies about keratinized tissues [4], we chose 80 Hz as the optimal frequency due to its differentiating capability (Text S1). For univariate analysis, the measured indices, *d*, *C*, *R_e_*, *Z*, and *θ*, at 80 Hz were standardized and/or logarithmized to be log *d*, *C_SD_*, *R_eSD_*, *Z_SD_*, log *Z_SD_* and *θ_SD_*, respectively. The distributional properties of these continuous variables were expressed by mean ± standard deviation (SD). These indices were then analyzed by fitting univariate logistic regression model with the generalized estimating equations (GEE) method for examination on the discrimination abilities between clavus and verruca data. Note that the use of the GEE method was to account for the correlations between the repeated measurements within subjects on the lesions.

To generate a best model with predictive factors, multivariate analysis was further performed by fitting multivariate logistic regression model with the same GEE method. The two indices, log *Z_SD_* and *θ_SD_*, were identified as predictive factors by a stepwise variable selection procedure. To validate the model, the basic model-fitting techniques were applied for (1) variable selection, (2) goodness-of-fit (GOF) assessment, and (3) regression diagnostics and remedies. The GOF assessments the estimated area under the receiver operating characteristic (ROC) curve, adjusted generalized *R* ^2^, and the Hosmer-Lemeshow GOF test. In practice, the value of the *c* statistic (0≤*c*≤1)≥0.7 suggests an acceptable level of discrimination power. Adjusted generalized *R* ^2^≥0.30 indicates an acceptable fit of a logistic regression model. And, larger *p* values of the Hosmer-Lemeshow GOF test indicate better fits. As regards to regression diagnostics, the non-linear effects of continuous covariates were detected by the generalized additive models (GAM), such that faults in the model can be exposed.

## Results

**Figure 1:**
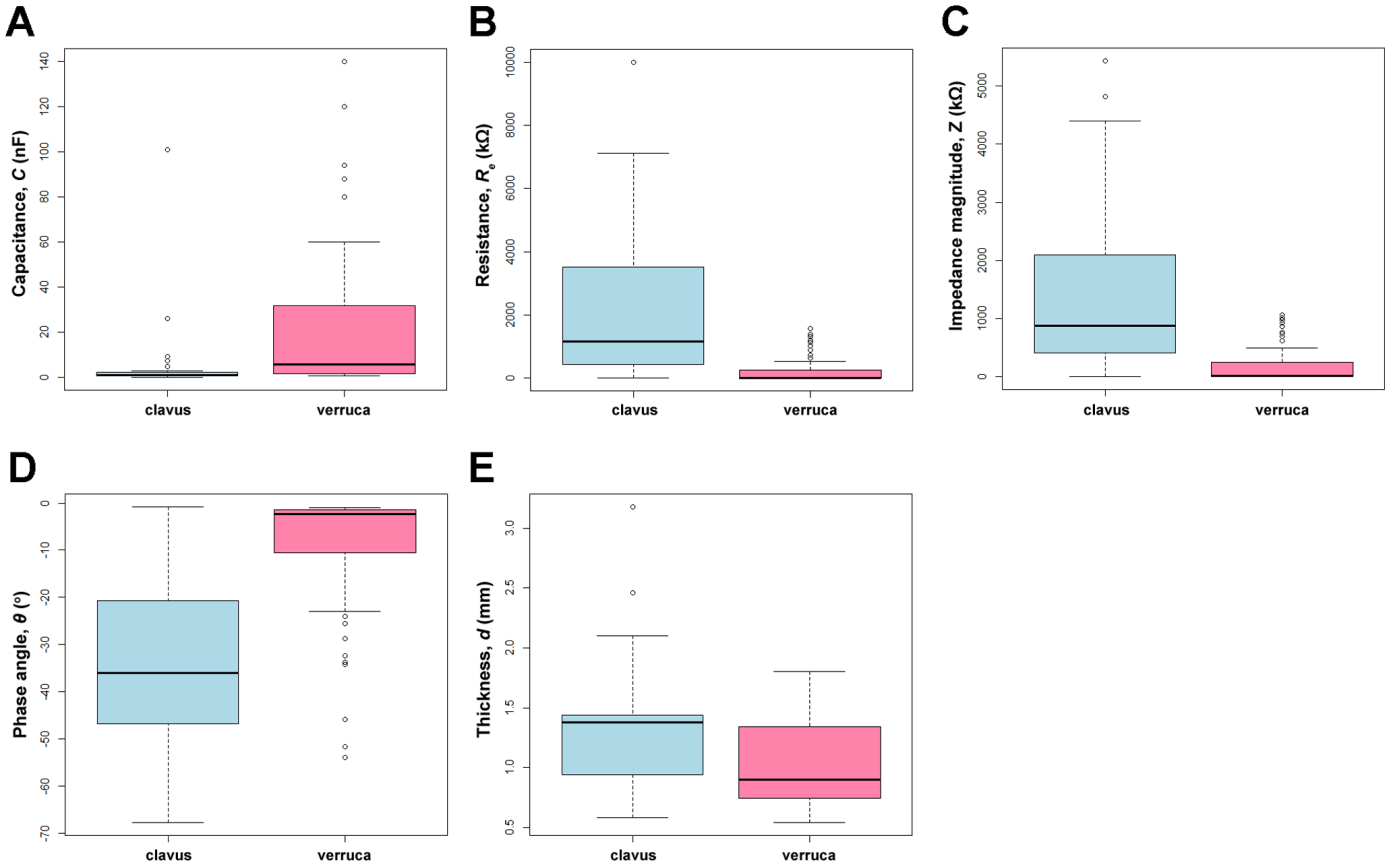
Conditional box plots of the all five measured indices, stratified by clavus and verruca. The lower edge, middle line, and upper edge of the box represent the 25^th^, 50^th^, and 75^th^ percentiles of the distribution of the measured index, at 80 Hz. The box plots, A, B, C, D, and E, are for capacitance (*C*), resistance (*R_e_*), impedance magnitude (*Z*), phase angle (*θ*) and thickness (*d*), respectively.

To discriminate the electrical properties between clavus and verruca, the measured and transformed indices of clavus at 80 Hz are compared with those of verruca in Table 1. For statistical analysis, 87 clavus and 84 verruca observations were used. By fitting univariate logistic regression analysis with the GEE method, all impedance indices are significantly different between clavus and verruca (*p*<0.001), except *C* and *C_SD_*. In Figure 1, the conditional box plots of the measured indices further demonstrate that the absolute value of *θ* index of clavus is higher than that of verruca. Moreover, *R_e_* and *Z* values of clavus are approximately ten times higher than those of verruca. These results indicate that high impedance is associated with clavus, whereas low impedance in verruca. Moreover, *R_e_* and *Z* indices can be used to best distinguish clavus and verruca.

**Table 1:** Comparison of impedance data between clavus and verruca at 80

	Clavus	Verruca	Total	*p* value
Number of subjects	15	14	29	
Number of lesions	29	28	57	
Number of observations	87	84	171	
**Measured variable:**				
*C* (nF)	4.21±15.31	19.85±28.66	11.84±24.07	0.099
	1.24 (0.17, 101)	5.85 (0.80, 140)	2.10 (0.17, 140.00)	
*R_e_* (kΩ)	1874.58±2095.43	209.02±397.98	1061.63±1734.63	<0.001
	1153.50 (1.90, 10000)	10.20 (0.74, 1559)	307.50 (0.74, 10000)	
*Z* (kΩ)	1280.19±1279.58	181.15±311.53	753.70±1094.39	<0.001
	873.90 (2.56, 5436)	16.24 (0.73, 1063)	357.30 (0.73, 5436)	
*θ* (^o^)	−33.71±18.36	−8.84±12.69	−21.80±20.16	<0.001
	−36.00 (−67.70, −0.81)	−2.36 (−53.90, −0.99)	−18.30 (−67.70, −0.81)	
*d* (mm)	1.34±0.55	1.02±0.36	1.19±0.49	<0.001
	1.38 (0.58, 3.18)	0.90 (0.54, 1.80)	1.10 (0.54, 3.18)	
**Transformed variable:**				
*C_SD_*	−0.06±0.85	0.80±1.59	0.36±1.33	0.100
	−0.23 (−0.29, 5.30)	0.03 (−0.25, 7.46)	−0.18 (−0.29, 7.46)	
*R_eSD_*	1.11±1.74	−0.27±0.33	0.43±1.44	<0.001
	0.51 (−0.45, 7.85)	−0.44 (−0.45, 0.85)	−0.19 (−0.45, 7.85)	
*Z_SD_*	1.13±1.61	−0.26±0.39	0.46±1.38	<0.001
	0.61 (−0.48, 6.35)	−0.46 (−0.48, 0.85)	−0.04 (−0.48, 6.35)	
*θ_SD_*	−0.33±0.88	0.87±0.61	0.24±0.97	<0.001
	−0.44 (−1.96, 1.25)	1.18 (−1.30, 1.24)	0.41 (−1.96, 1.25)	
log *Z_SD_*	1.02±0.75	−0.43±1.02	0.32±1.15	<0.001
	1.19 (−1.41, 2.01)	−0.58 (−1.96, 1.28)	0.79 (−1.96, 2.01)	
log *d*	0.22±0.39	−0.04±0.34	0.09±0.39	<0.001
	0.32 (−0.54, 1.16)	−0.11 (−0.62, 0.59)	0.10 (−0.62, 1.16)	

Notes: The measured variables, *d*, *C*, *R_e_*, *Z*, and *θ*, indicate thickness, capacitance, resistance, impedance magnitude, and phase angle, respectively. The transformed variables, *C_SD_*, *R_eSD_*, *Z_SD_*, and *θ_SD_*, signify standardized *C*, *R_e_*, *Z*, and *θ* values, respectively. The transformed variables, log *d* and log *Z_SD_* denote logarithmized *d* value and standardized logarithmized *Z* value. The listed values were mean ± standard deviation (SD) on the upper row and median (range) on the lower one. All *p*-values of group comparisons are obtained by fitting univariate logistic regression models with the generalized estimating equations (GEE) method to account for the correlations between repeated measurements.

**Figure 2:**
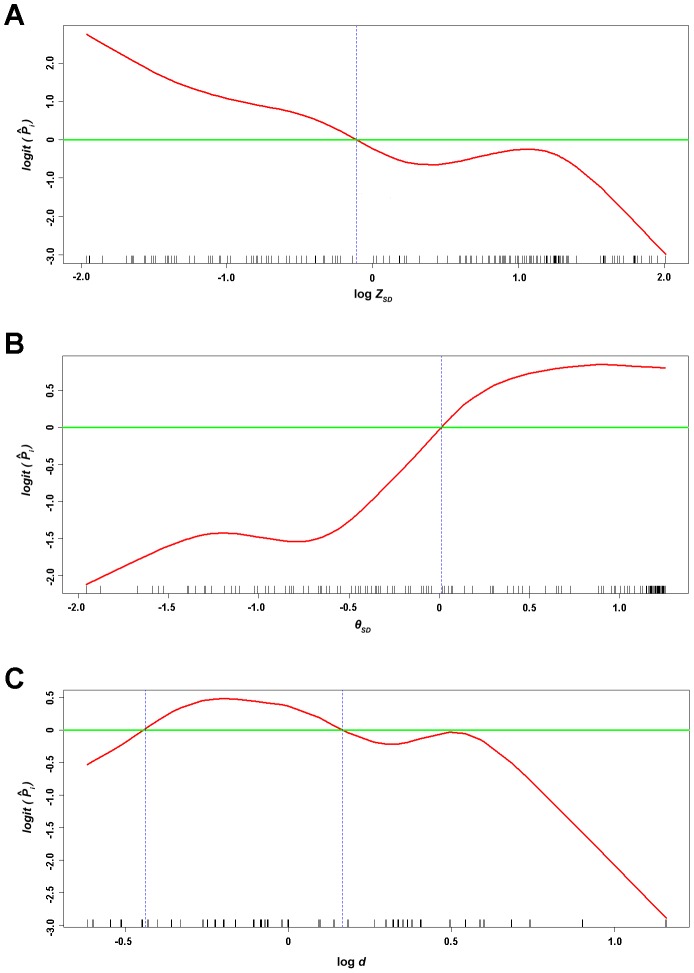
The GAM plots of the predictors, log *Z_SD_* (A), θ*_SD_* (B), and log *d* (C) respectively. The generalized additive models plots reveal the smoothed partial effects of the predictors in modeling the probability of being verruca. The distribution of the observed values of log *Z_SD_*, *θ_SD_*, and log *d* are shown by the rugs on the *X*-axes. The *Y*-axes are the *logit* of the estimated probability of being verruca (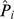), i.e., log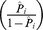. The horizontal green line indicates the place where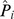 = 0.5.

To generate a model for estimating the probability of being verruca versus clavus, all the indices, are analyzed using multivariate logistic regression with the GEE method. Moreover, the GAM plots of Figures 2A and 2B depict the approximately linear partial effects of predictors, log *Z_SD_* and *θ_SD_*, for the probability of being verruca respectively, but Figure 2C reveals an apparently nonlinear partial effect of log *d* and identified two appropriate cut-off points in discretizing. Therefore, one of the best models is selected (Text S2). In the final model, the estimated odds ratios (ORs) of predictors, log *Z_SD_* and *θ_SD_*, are 0.406 (*p* = 0. 1121) and 2.304 (*p* = 0.2406), respectively (Table 2). The probability of being verruca (i.e., the predicted value) can be estimated by
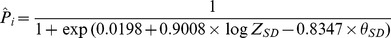



**Table 2:** Multivariate analysis of the predictors of verruca at 80(GEE) method.

Covariate	Estimate regression coefficient	Robust Standard Error	Chi-Square test	*p* Value	Estimated Odds Ratio	95% Confidence Interval of Odds Ratio
Intercept	−0.0198	0.5613	0.0012	0.9719	-	-
log *Z_SD_*	−0.9008	0.5670	2.5245	0.1121	0.406	0.134-1.234
*θ_SD_*	0.8347	0.7112	1.3773	0.2406	2.304	0.572-9.288

Goodness-of-fit assessment: Number of clusters  = 57, number of observations  = 166, the estimated area under the Receiver Operating Characteristic (ROC) curve  = 0.875>0.7, adjusted generalized *R* ^2^ = 0.512>0.3, and Hosmer-Lemeshow goodness-of-fit *F* test *p* = 0.350>0.05 (df = 9, 156).

Prediction: To calculate the estimated probability of being verruca (i.e., the *predicted value*,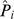) given the observed covariate values, one can use the following formula. According to the above fitted multiple logistic regression model

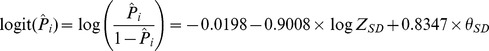
the *predicted value* of observation *i* is

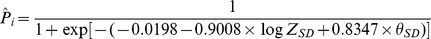
where log *Z_SD_* =  logarithmized standardized *Z* value, and θ*_SD_* =  standardized θ value.

**Figure 3:**
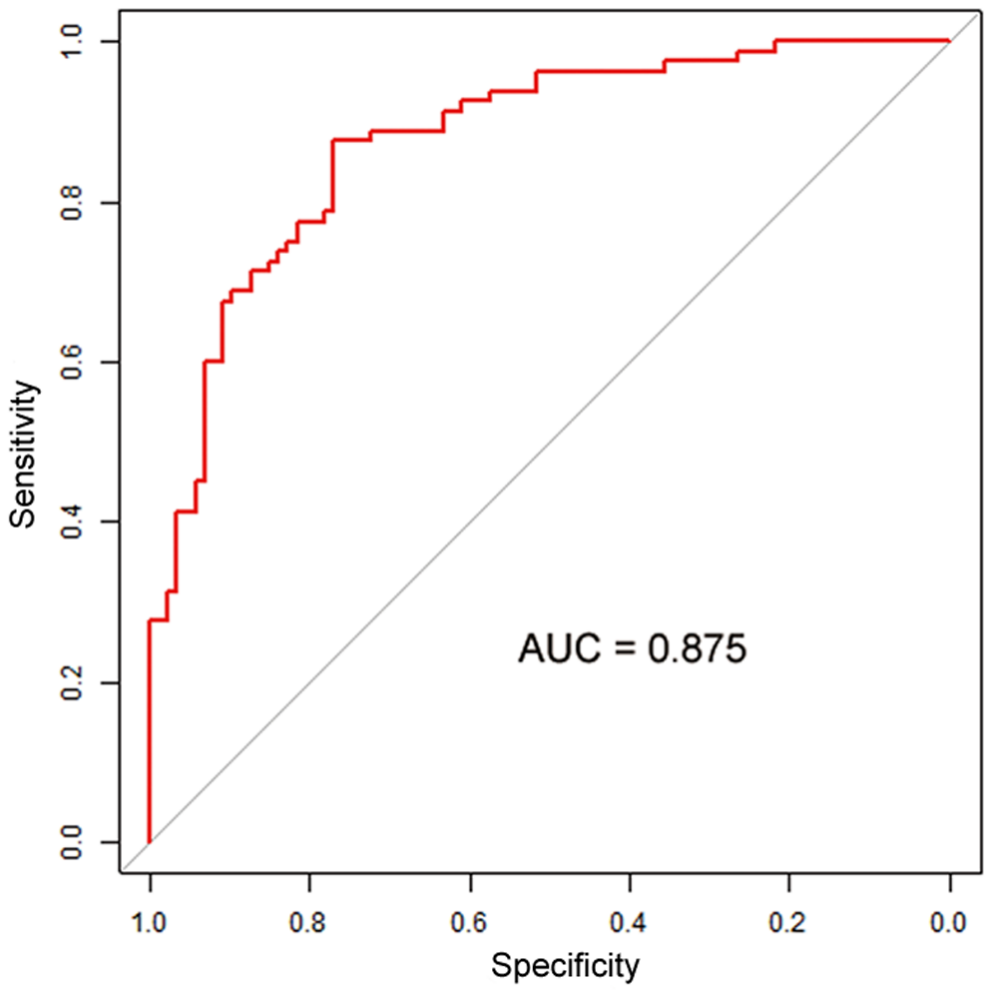
The Receiver Operating Characteristic (ROC) curve for the prediction of verruca. The estimated area under the ROC curve (AUC) is 0.875.

As 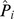 value approaches 1, the more probable the sample is associated with verruca. If 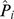 value approaches 0, the more likely the sample is clavus. Furthermore, based on the above model, GOF assessment is used to validate the performance of the model. The area under the ROC is 0.875 (>0.7) (Figure 3), the adjusted generalized *R* ^2^ is 0.512 (>0.3), and the *p* value of the Hosmer-Lemeshow GOF test is 0.350 (>0.05). These results all pass the tests for best model-fitting techniques. Overall, a model with log *Z_SD_* and *θ_SD_* as predictive factors is generated for differentiating verruca from clavus, and the validity of the model is determined by GOF method.

## Discussion

Impedance is proposed in this study to reflect the different histological constituents of clavus and verruca, so as to assemble a new electrical impedance system for differentiating these two lesion types. Research have disclosed that the impedance/resistivity of stratum corneum is greater than those of other tissues in the human body [24], [25], whereas hydration of intact skin decreases the impedance [10], [11]. In other words, impedance is high in stratum corneum and correlates reversely with the degree of hydration and admittance. Therefore, the relative high impedance values of clavus lesion found in the study may be resulted from its massive hyperkeratosis and relative thin, atrophic epidermis, which in turn point to the translucent central core under a dermoscope and biopsies. On the contrary, the components of verruca-relative thin stratum corneum, acanthosis proliferated by keratinocytes, and capillary proliferation-may explain why low impedance values are measured. Such differences in impedance values elucidate electrical impedance to be a competitive modality for discriminating the heterogeneous components of clavus from those of verruca.

To identify predictive factor from electrical impedance for model generation, this study discerns statistic differences between clavus and verruca, regarding resistance, impedance magnitude, phase angle, and thickness, but not capacitance. More importantly, resistance and impedance magnitude between clavus and verruca can differ by a factor of ten. However, only the model generated from the two parameters-impedance magnitude and phase angle-fits the data most. This can be explained by the following two equations [18]:
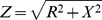


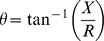



Here, impedance is defined as total resistance of a biological conductor on alternating current. Simultaneously, it is comprised of two parts: one is resistance, and the other is reactance, defined as *X*. The reason why impedance magnitude and phase angle are the two parameters for model generation is clear. Furthermore, the advantage of using these two parameters is that they are transformed and calibrated values and as a result of that the predictive procedure becomes far less complicated. It is worthy to note that the use of these two indices in model design has been revealed in previous research, though relating neither to clavus nor to verruca. Impedance magnitude is found to be one of the important indices in analyzing electrical impedance spectra [6], [10], [12], [18]–[20], [26], [27]. While, phase angle is in association with the studies concerning cancers, chronic obstructive pulmonary disease, HIV, hospital mortality of geriatric patients, and skin condition test [10], [12], [26], [28], [29].

After discussion on the three indices in question, attention is driven to the parameters, thickness and capacitance. Thickness parameter, although showing capabilities in distinguishing between clavus and verruca, is observed to pose problems in impedance analysis, due partly to the difficulties in specifying measured locations, and due partly to its variations caused by biological factors and environmental conditions [10], [27]. In addition to this, the thickness of the sample, unlike all the other indices acquired by LCR-meter, is measured manually. Therefore, a model generated without thickness as predictor not only eliminates uncertainties embedded within, but also simplifies its procedure. As for capacitance, it is the only index derived from electrical impedance delivers otherwise result.

Last but not the least, the limitations of the model are detected by the GAM plots. Theoretically, the plots should be linear, but a partial linear is plotted. The non-linear effects of continuous covariates expose the inadequacies of the model. That is to say, misdiagnosis may occur, when lesions are not typical. Non-typical lesions include verruca with very thick corneal layer and few capillary proliferation, and clavus with exceeding hydration.

## Conclusions

In conclusion, we employed electrical impedance as a novel tool to postulate a predictive model for differential diagnosis of clavus and verruca. From electrical impedance, log *Z_SD_* and *θ_SD_*, were two predictive factors derived for estimating the probability of being verruca versus clavus. Moreover, the validity of the model was approved by GOF and GAM methods. In spite of certain limitations, this study provides a rapid, low cost, safe and non-invasive alternative modality for physicians' clinic practice. Further application on *in vivo* diagnosis and investigation on circuit design for size reduction are expected.

## Supporting Information

Figure S1 **The stereoscopic features of (A) clavus and (B) verruca.**
(PDF)

Text S1 **Frequency selection.**
(PDF)

Text S2 **Final model selection.**
(PDF)
