# Supplementary figures and images for: In Vitro Differential Diagnosis of Clavus and Verruca by a Predictive Model Generated from Electrical Impedance
Source: PLoS One. 2014 Apr 4;9(4):e93647. doi: 10.1371/journal.pone.0093647 (PMC3976310; doi:10.1371/journal.pone.0093647)

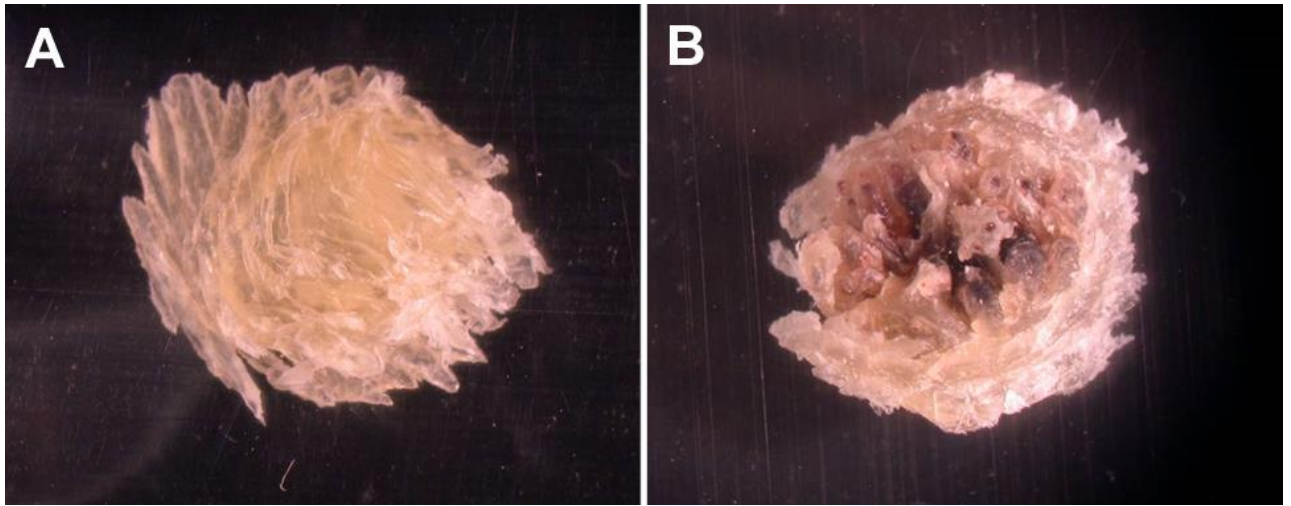

**Figure S1.** The stereoscopic features of (A) clavus and (B) verruca.

Supplement: Figure S1 — The stereoscopic features of (A) clavus and (B) verruca. (PDF) [file pone.0093647.s001.pdf]
