# Supplementary material for: In Vitro Differential Diagnosis of Clavus and Verruca by a Predictive Model Generated from Electrical Impedance
Source: PLoS One. 2014 Apr 4;9(4):e93647. doi: 10.1371/journal.pone.0093647 (PMC3976310; doi:10.1371/journal.pone.0093647)

Supplementary Text S1

Frequency selection

The data were collected at different frequencies in our experiment, but 80 Hz was chosen for maximizing the discriminating powers of studied variables on disease diagnosis. Specifically, as shown in the figure below, conditioning scatter plots (called the 'coplots') were drawn to show the ability in differentiation of binary responses by each studied variable at 10 different frequencies (Hz) and another controlling variable including thickness, duration, relative humidity, and environmental temperature. The frequencies at 50 Hz, 80 Hz, and 100 Hz yielded essentially the same results. We arbitrarily chose the middle one, 80 Hz, and considered it optimal for our data analysis.

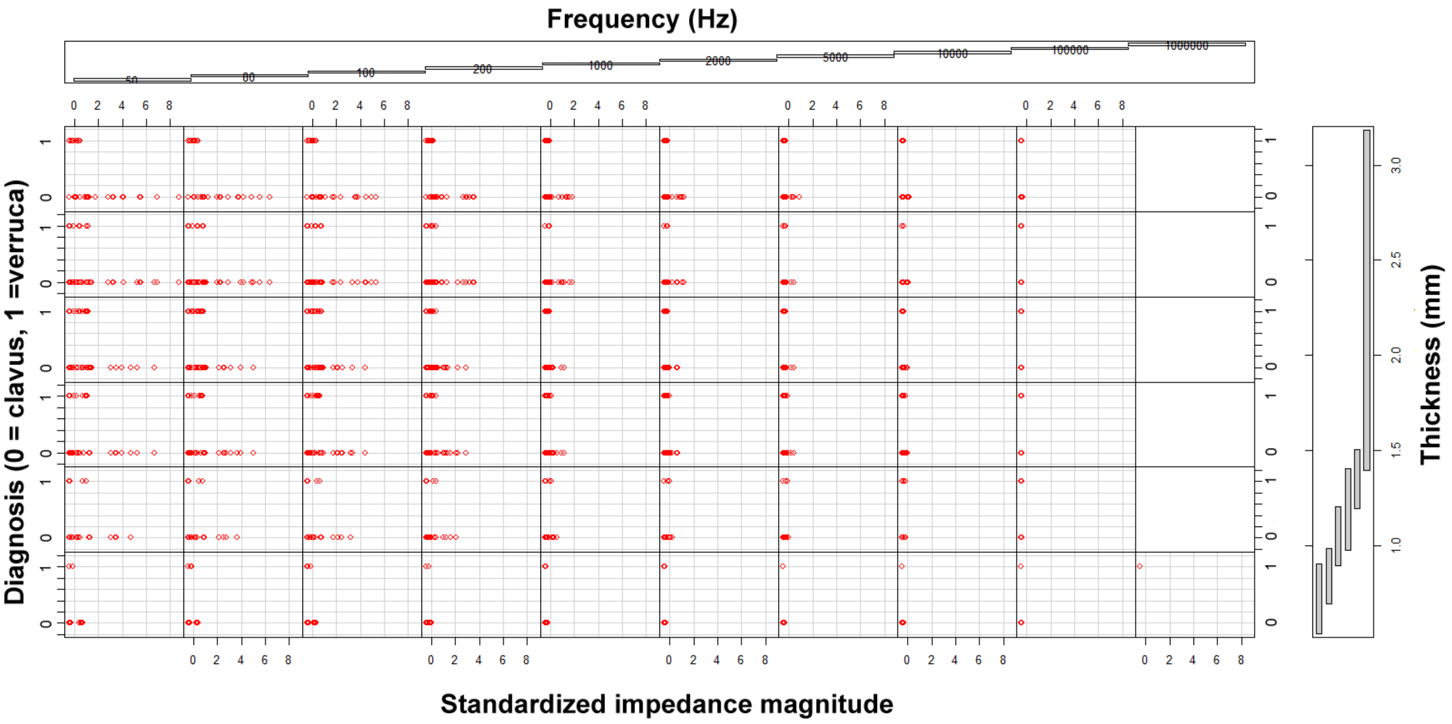

Supplement: Text S1 — Frequency selection. (PDF) [file pone.0093647.s002.pdf]
