# Supplementary material for: In Vitro Differential Diagnosis of Clavus and Verruca by a Predictive Model Generated from Electrical Impedance
Source: PLoS One. 2014 Apr 4;9(4):e93647. doi: 10.1371/journal.pone.0093647 (PMC3976310; doi:10.1371/journal.pone.0093647)
Supplement: Text S2 — Final model selection. (PDF) [file pone.0093647.s003.pdf]

Supplementary Text S2

Final model selection

Multivariate analysis was performed by fitting GEE logistic regression models of diagnosis (0 = clavus, 1 = verruca) with all measured and transformed impedance indices at 80 Hz and thickness. Moreover, the GAM plots of Figures 2A and 2B depicted the approximately linear partial effects of log Z_{SD} and θ_{SD} on the logit of the probability of being verruca respectively, but the GAM plot of Figure 2C revealed an apparently nonlinear partial effect of log d and identified two appropriate cut-off points, -0.439 and 0.166, for discretizing log d to maximize its discriminating power. In the table below (Table S1), we listed the five candidates of final GEE logistic regression models obtained from the stepwise variable selection procedures. Among them, **Model 5** was chosen as our final GEE logistic regression model for differential diagnosis of clavus and verruca due to the following four reasons:

1. Both log Z_{SD} and θ_{SD} alone were strong predictors for differential diagnosis — especially log Z_{SD} according to the difference in the value of the c statistic between **Models 1** and **2**.
2. As shown in **Model 4**, thickness had also a contribution to prediction if the discretized log d, i.e., 1 = log $d \geq$ -0.439 and log d < 0.166 and 0 = otherwise (Figure 2C), was added to **Model 2**, but not true if the same discretized log d was added to **Model 1**.
3. Nevertheless, **Models 2** and **4** did not pass the Hosmer and Lemeshow goodness-of-fit F test (i.e., $p < 0.05$).

4. Hence, **Model 5** became the best one by including both log Z_{SD} and θ_{SD} as the predictors for differential diagnosis. It had the second highest value of the *c* statistic (0.875) among those five GEE logistic regression models, which was very close to the highest value of the *c* statistic (0.879). As compared to **Models 1** and **2** respectively, the reduction in the values of estimated regression coefficients and the inflation in the values of the estimated robust standard errors were caused by the high correlation between log Z_{SD} and θ_{SD} (Pearson correlation = -0.840 and Spearman rank correlation = -0.856), but log Z_{SD} and θ_{SD} complemented each other in diagnosis of clavus versus verruca.

As listed in Table S1, the final GEE logistic regression model of diagnosis (0 = clavus, 1 = verruca) (**Model 5**) was

$$\text{logit}(\hat{P}_i) = \log\left(\frac{\hat{P}_i}{1-\hat{P}_i}\right) = (-0.0198) - 0.9008 \times \log Z_{SD}) + 0.8347 \times \theta_{SD}$$

and the estimated probability of being verruca (i.e., the *predicted value*) for observation *i* could be calculated by

$$\hat{P}_i = \frac{1}{1+\exp\left[-\left(-0.0198 - 0.9008 \times \log(Z_{SD}) + 0.8347 \times \theta_{SD,}\right)\right]}$$

accordingly. The larger the predicted value $\hat{P}_i$, the more likely the lesion was verruca.

Specifically, log Z_{SD} (estimated odds ratio = exp(-0.9008) = 0.406), p = 0.1121) had a negative effect and θ_{SD} (estimated odds ratio = exp(0.8347) = 2.304), p = 0.2406) had a positive effect on the probability of being verruca. As mentioned before, the relatively high correlation

between log Z_{SD} and θ_{SD} (Pearson correlation = -0.840 and Spearman rank correlation = -0.856) caused the reduction in the values of estimated regression coefficients and the inflation in the values of the estimated robust standard errors, and thus the two inflated *p* values did not truly reflect the strengths of the evidences against the null hypotheses. More importantly, **Model 5** fitted the observed data quite well. The area under the ROC curve was 0.875 > 0.7 (Figure 3), the adjusted generalized R^2 was 0.512 > 0.3, and the *p* value of the Hosmer-Lemeshow GOF test was 0.350 > 0.05. Thus, **Model 5** was a sound choice for the purpose of prediction.

References

1. Højsgaard S, Halekoh U, Yan J (2006). The R package geepack for generalized estimating equations. J Stat Softw 15(2): 1-11.
2. Liang KY, Zeger SL (1986). Longitudinal data analysis using generalized linear models. Biometrika 73: 13-22.
3. Prentice RL, Zhao LP (1991). Estimating equations for parameters in means and covariances of multivariate discrete and continuous responses. Biometrics 47: 825-839.
4. Yan J, Fine JP (2004). Estimating equations for association structures. Stat Med 23: 859-880.
5. Yee TW (2013). VGAM: Vector generalized linear and additive models. R package, version 0.9-2 (URL: http://CRAN.R-project.org/package=VGAM).
6. Yee TW, Wild CJ (1996). Vector generalized additive models. J R Stat Soc Series B Stat

Methodol 58(3): 481-493.

Table S1. Multivariate analyses of the characteristics of the lesions for diagnosis of verruca at 80 Hz by fitting multiple logistic regression models with the generalized estimating equations (GEE) method

Covariate	Estimated regression coefficient	Robust standard error	Chi-square test	*p* value	Estimated odds ratio	95% confidence interval of odds ratio
**Model 1:**[1]						
Intercept	0.5099	0.4073	1.5668	0.2107	—	—
$\log(Z_{SD})$	-1.5317	0.3710	17.0433	< 0.0001	0.216	0.104 − 0.447
**Model 2:**[2]						
Intercept	-0.7098	0.4148	2.9281	0.0870	—	—
θ_{SD}	1.7987	0.4665	14.8644	0.0001	6.042	2.421 − 15.077
**Model 3:**[3]						
Intercept	0.0557	0.5375	0.0108	0.9174	—	—
$\log Z_{SD}$	-1.4930	0.3967	14.1605	0.0002	0.225	0.103 − 0.489
$-0.439 \leq \log d < 0.166$	1.1147	0.7089	2.4728	0.1158	3.049	0.760 − 12.234
**Model 4:**[4]						
Intercept	-1.3654	0.5246	6.7750	0.0092	—	—
θ_{SD}	1.8133	0.4641	15.2625	< 0.0001	6.130	2.468 − 15.225
$-0.439 \leq \log d < 0.166$	1.6032	0.7429	4.6564	0.0309	4.969	1.158 − 21.314
**Model 5 (Final Model):**[5]						
Intercept	-0.0198	0.5613	0.0012	0.9719	—	—
$\log(Z_{SD})$	-0.9008	0.5670	2.5245	0.1121	0.406	0.134 − 1.234
θ_{SD}	0.8347	0.7112	1.3773	0.2406	2.304	0.572 − 9.288

1. **Goodness-of-fit assessment:** Number of clusters = 57, number of observations = 166, the estimated area under the Receiver Operating Characteristic (ROC) curve = 0.864 > 0.7, adjusted generalized $R^2 = 0.492 > 0.3$, and the Hosmer-Lemeshow goodness-of-fit *F* test $p = 0.336 > 0.05$ (df = 9, 156).
2. **Goodness-of-fit assessment:** Number of clusters = 57, number of observations = 166, the estimated area under the Receiver Operating Characteristic (ROC) curve = 0.853 > 0.7, adjusted generalized $R^2 = 0.477 > 0.3$, and the Hosmer-Lemeshow goodness-of-fit *F* test $p = 0.012 < 0.05$ (df = 9, 156).
3. **Goodness-of-fit assessment:** Number of clusters = 57, number of observations = 166, the estimated area under the Receiver Operating Characteristic (ROC) curve = 0.875 > 0.7, adjusted generalized $R^2 = 0.527 > 0.3$, and the

Hosmer-Lemeshow goodness-of-fit F test $p = 0.342 > 0.05$ (df = 9, 156).

4. **Goodness-of-fit assessment:** Number of clusters = 57, number of observations = 166, the estimated area under the Receiver Operating Characteristic (ROC) curve = 0.879 > 0.7, adjusted generalized $R^2 = 0.549 > 0.3$, and the Hosmer-Lemeshow goodness-of-fit F test $p = 0.017 < 0.05$ (df = 9, 156).
5. **Goodness-of-fit assessment:** Number of clusters = 57, number of observations = 166, the estimated area under the Receiver Operating Characteristic (ROC) curve = 0.875 > 0.7, adjusted generalized $R^2 = 0.512 > 0.3$, and the Hosmer-Lemeshow goodness-of-fit F test $p = 0.350 > 0.05$ (df = 9, 156).

- **Prediction:** To calculate the estimated probability of being verruca (i.e., the *predicted value*, $\hat{P}_i$) given the observed covariate values, one can use the following formula. According to the above fitted final GEE logistic regression model (i.e., **Model 5**),

$$\text{logit}(\hat{P}_i) = \log\left(\frac{\hat{P}_i}{1-\hat{P}_i}\right) = (-0.0198) - 0.9008 \times \log(Z_{SD}) + 0.8347 \times \theta_{SD}$$

the *predicted value* of observation i is

$$\hat{P}_i = \frac{1}{1 + \exp\left[-\left(-0.0198 - 0.9008 \times \log Z_{SD}\right) + 0.8347 \times \theta_{SD}\right)\right]}$$

where $\log Z_{SD}$ = logarithmized standardized Z value and θ_{SD} = standardized θ value.
